# Methods to Study the Myenteric Plexus of Rat Small Intestine

**DOI:** 10.1007/s10571-021-01181-5

**Published:** 2021-12-21

**Authors:** Ines Hecking, Lennart Norman Stegemann, Sarah Stahlke, Verena Theis, Matthias Vorgerd, Veronika Matschke, Carsten Theiss

**Affiliations:** 1 Department of Cytology, Institute of Anatomy, Ruhr-University Bochum, 44801 Bochum, Germany; 2 Department of Neurology, Neuromuscular Center Ruhrgebiet, University Hospital Bergmannsheil, 44789 Bochum, Germany

**Keywords:** Enteric nervous system, Laser microdissection, Enteric ganglia, Gut, Myenteric plexus cell culture

## Abstract

The close interaction between the enteric nervous system, microbiome, and brain in vertebrates is an emerging topic of recent studies. Different species such as rat, mouse, and human are currently being used for this purpose, among others. The transferability of protocols for tissue isolation and sample collection is not always straightforward. Thus, the present work presents a new protocol for isolation and sample collection of rat myenteric plexus cells for in vivo as well as in vitro studies. With the methods and chemicals described in detail, a wide variety of investigations can be performed with regard to normal physiological as well as pathological processes in the postnatal developing enteric nervous system. The fast and efficient preparation of the intestine as the first step is particularly important. We have developed and described a LIENS chamber to obtain optimal tissue quality during intestinal freezing. Cryosections of the flat, snap-frozen intestine can then be prepared for histological examination of the various wall layers of the intestine, e.g. by immunohistochemistry. In addition, these cryosections are suitable for the preparation of defined regions, as shown here using the ganglia of the mesenteric plexus. This specific tissue was obtained by laser microdissection, making the presented methodology also suitable for subsequent analyses that require high quality (specificity) of the samples. Furthermore, we present here a fully modernized protocol for the cultivation of myenteric neurons from the rat intestine, which is suitable for a variety of in vitro studies.

**Supplementary Information:**

The online version contains supplementary material available at 10.1007/s10571-021-01181-5.

## Introduction

The enteric nervous system (ENS), also referred to as the second brain due to its similarity to the CNS in terms of complexity, size and expressed neurotransmitters, is able to control elaborated processes in the gastrointestinal tract (GIT) largely autonomously (Gershon 1998). Motility, absorption, blood flow and secretion are regulated by several hundred million neurons and glial cells organized in two ganglionic plexuses in the wall of the GIT. The myenteric ganglia are organized as nerve plexuses between the longitudinal and circular smooth muscle layers, called the tunica muscularis, and extend throughout the entire digestive tract in mammals. The second plexus is formed in the tunica submucosa (Furness 2000; Kunze and Furness 1999).

Enteric nerve cells are able to adapt their functions appropriately across the lifespan in interaction with other cell types of the ENS and in response to stimuli such as mechanical stretching or inflammatory bowel diseases (Schäfer et al. 2009). While mechanical stretching leads to an increased expression of immediate early genes in myenteric neurons of rats (Dimaline et al. 1995), cytokines released during inflammatory bowel diseases alter the transmitter release in the ENS (Schäfer et al. 2009). Furthermore, in case of such an inflammation, neurons of the myenteric plexus show changes both in the size of synaptic vesicles and in their recycling rate (Krauter et al. 2007). The neurons, in turn, probably promote the activation of the immune system via the local release of neuropeptides such as substance P (Collins 1996; Furness 2000; Furness et al. 1999). Nutrients and their influence on the phenotype and functionality of the ENS are also subject of current studies (Neunlist and Schemann 2014). Despite its autonomous function, there is a close connection between the ENS and the central nervous system (CNS) via sympathetic, parasympathetic and endocrine signal transmission called the gut-brain axis. These bidirectional connections may explain how physiological and pathological stimuli in the ENS affect the CNS (Mayer 2011). Recently, it has been suggested that the influence of the gut microbiota on CNS development and functionality may also lead to pathological changes in adults, as those associated with the development of functional bowel diseases (Mayer et al. 2014). A largely dysfunctional gut-brain axis is also discussed in connection with the pathogenesis of neurodegenerative diseases, like as Parkinson's and Alzheimer´s disease, in which, in addition to increased permeability due to inflammation, altered microbiota via the nervus vagus presumably also lead to changes in the CNS (Chapelet et al. 2019; Kim et al. 2019; Köhler et al. 2016).

However, the interactions between the ENS and CNS are far from fully understood, so further studies are urgently needed. To investigate individual cellular aspects of this interaction, e.g. in a direct experimental approach in vivo and in vitro*,* the portfolio of methods for isolating ENS cells needs to be expanded. A simple method for isolating specific cells or cell groups from in vivo tissue material is laser microdissection (LMD). Several studies have already shown that highly specific intestinal ganglia can be harvested to isolate high-quality RNA from human intestinal sections for subsequent cDNA synthesis and real-time quantitative PCR (RT-qPCR) (Böttner et al. 2010; May-Zhang et al. 2018). However, the enormous size difference of the GIT in humans compared to smaller model animals such as the rat requires numerous adaptations in sample collection and cultivation. Therefore, in the following protocol, based on the work of May-Zhang et al. (2018) we describe numerous modifications to prepare the GIT of small animals, like the rat, for ENS studies. The protocol includes several adjustments and optimizations to the work of May-Zhang et al. (2018) in order to be able to produce suitable sections for both immunohistochemical studies and LMD with, e.g. subsequent PCR studies. In addition, the described protocol can be used to perform relatively simple in vitro studies on ENS neurons with the possibility of direct experimental manipulation. Such protocols are necessary to provide new models for physiological, electrophysiological and pharmacological studies (Grundmann et al. 2015). Although the basic principle of ENS cell cultures was already described by Yaffe (1968), materials and techniques developed in recent years have helped to significantly improve older protocols, e.g. improving the composition of the growth medium to increase the survival rate and reduce the dedifferentiation of cells in the culture (Willard and Nishi 1985; Grundmann et al. 2015).

## Materials and Methods

All experiments in this study have been performed in strict accordance with institutional, German (TschG), and EU guidelines for the care and use of laboratory animals.

### Preparation of the Rat Small Intestine for Cryosectioning

**Fig. 1:**
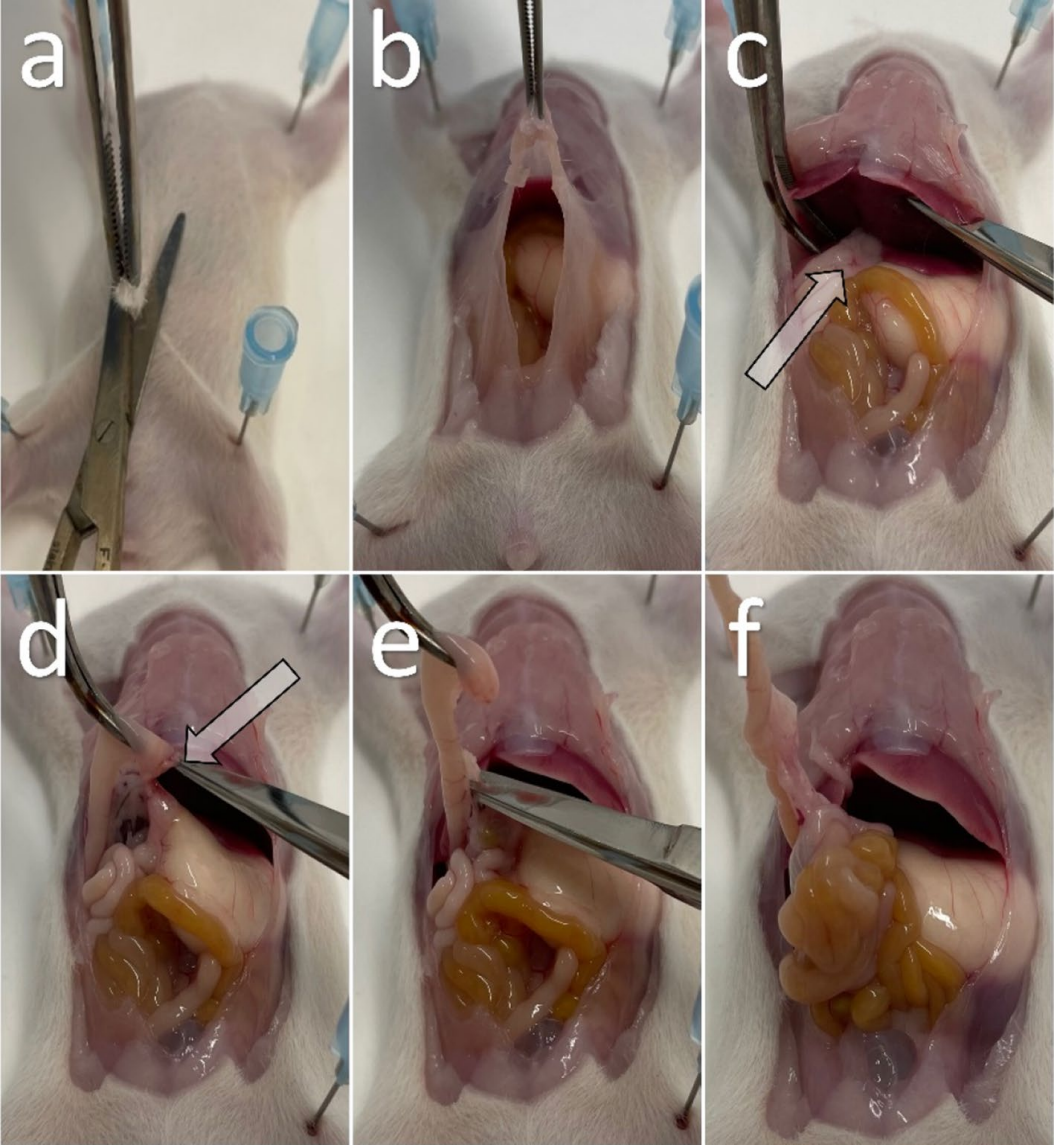
**a** A decapitated Wistar rat is fixed on a polystyrene plate and prepared for removal of the peritoneum. **b** The abdomen and peritoneum are opened. **c** The arrow marks the junction of the pylorus and duodenum below the right liver. **d** The arrow shows a cut distal to the pylorus with the mesentery still attached to the intestine. **e** The intestine is carefully detached from its mesentery. **f** The extracted bowel is mostly free of mesentery

All solutions are prepared with DEPC-treated water (D5758-50ML). The work surfaces are cleaned with NaOH-EDTA (0.1MNaOH, 1 mM EDTA). Preparation tools and petri dishes are sterilized for 4 h at 240 °C. Male and female Wistar rats are decapitated at postnatal day 0 (p0), 9 (p9), 15 (p15), and 30 (p30). After p0, animals were previously anesthetized with chloroform. The rat is fixated with needles on a polystyrene plate to facilitate the removal of the intestine (Fig. 1 and supplement movie 1). First, only the skin with fur is removed with large scissors and forceps (Fig. 1a), and then the peritoneum is opened with new and clean tools (Fig. 1b). The intestine is separated just below the stomach (Fig. 1c,d), and then carefully detached from the mesentery with a small scissor (Fig. 1e). In the next step, the entire length of the small intestine is removed from the mesentery by carefully pulling it out through its own intestinal loops (Fig. 1f). The removed intestine is cut into two or three pieces, depending on the length, and stored in a petri dish with cold and RNase-free PBS. After flushing the intestine with cold (RNase-free) PBS using a syringe, it is transferred to a new petri dish with clean and cooled PBS. In the last step, the intestine is opened lengthwise along the mesentery and cut into 2–4 cm-long pieces with fine scissors to unfold it for further processing.

### Freezing for Further Studies

**Fig. 2:**
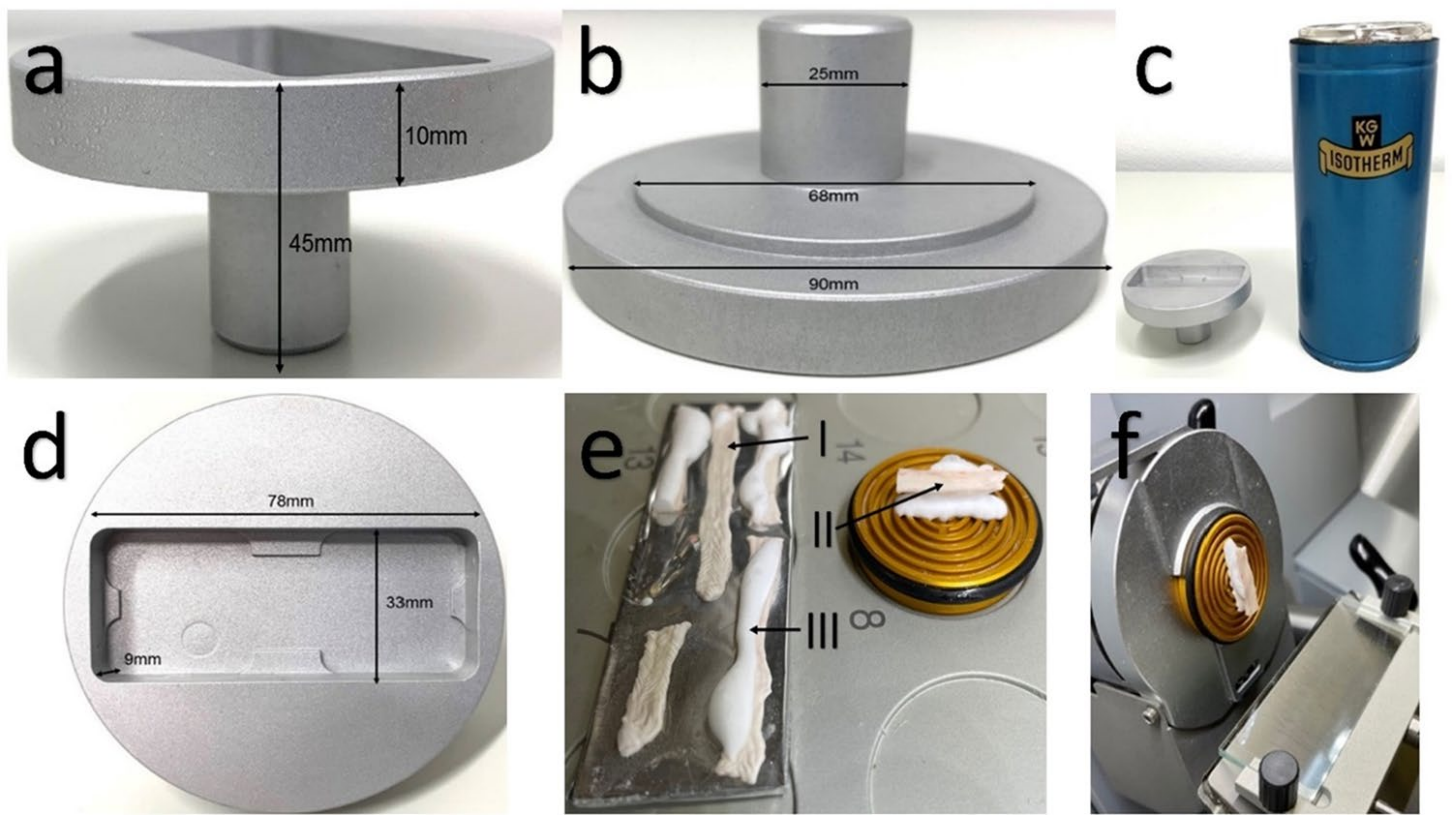
**a** Side view of the LIENS chamber made of aluminium for quick freezing of specimens. The chamber was developed to reduce the use of liquid nitrogen and to provide a very controlled and uniform freezing process. **b** The outer ring of the LIENS chamber has a diameter of 90 mm, while the middle ring is 68 mm wide to be accommodated by a thermos flask. The diameter of the PTC thermistor is 25 mm. **c** LIENS chamber next to thermos flask filled with liquid nitrogen. **d** Slide freezer holder is 35 mm × 78 mm wide and 9 mm deep and contains 4 × 1 mm bars. **e** Frozen tissue on the slide inside the cryostat, I: Mucosal side of the intestine; II: Serosal side of the intestine; III: Intestinal pieces are stabilized on the mucosal side with tissue freezing medium and then frozen on the specimen holder, so the serosal side points to the top. **f** Clamped specimen holder in the CryoStar NX50, perfectly aligned for a smooth cut

Microscope slides (Thermo Scientific, AAAA000001##12E) covered with aluminium foil are sterilized for 4 h at 240 °C before they are used as a base for freezing. The 2–3 cm long pieces of intestine are carefully blotted onto a stack of Kimtech (Kimberly-Clark Professional) and lightly dried. Immediately afterwards, 2–3 pieces of intestine at a time are carefully spread out on the prepared slide with the serosa facing downwards, without stretching the tissue. It is important that the tissue lies completely flat and that no air bubbles are trapped underneath in order to achieve a maximum area of ganglia per section in the further steps. The homemade LIENS chamber (Fig. 2a–d) is designed to ensure that the entire slide is frozen quickly and evenly. It is first stored at − 20 °C, and then placed on a vessel filled with liquid nitrogen for cooling to − 50 °C for preparation. Isopentane is poured into the bulge for the slide. The slides are placed in isopentane with tweezers as soon as the isopentane has reached a temperature of − 50 °C.

Once the tissue is completely frozen, the slide is transferred from the LIENS chamber to an open cuvette at − 20 °C. After the isopentane on the slides has evaporated, two slides are stored back to back in a 50 ml tube (62.547.254, Sarstedt) at − 20 °C for immunohistochemistry or at − 80 °C for LMD. The described protocol should be performed by two persons at the same time, so that the entire preparation including freezing of the material can be performed within 15 min per rat.

### Cryosections of Rat Intestine

First, the removable parts of the cryostat (CryoStar NX50, Thermo Scientific) are cleaned with NaOH-EDTA (0.1MNaOH, 1 mM EDTA). In the cryostat, the frozen tissue is tempered at a chamber temperature of − 20 °C and a stage temperature of − 18 °C to − 16 °C for 45 min. Subsequently, each piece of intestine is stabilized with a strip of tissue freezing medium (#14020108926, Leica Biosystems) to prevent deformation during detachment; the serosa points downwards (Fig. 2e-I, e-III). By adjusting the specimen holder very precisely, the individual tissue layers are cut straight in one plane, starting with the serosa and then followed by the muscularis with the myenteric plexus (Fig. 2f). From time to time the unstained sections are examined by light microscopy to ensure correct alignment. The greatest number of ENS ganglia is found in sections containing cells from both the longitudinal and the circular muscle layers. Depending on age, the transition from the longitudinal to the circular muscle layer occurs after a maximum of 10 sections of 10 µm thickness, followed by about 10 useful sections (10 µm thickness) with many neurons detectable down to the submucosa. Depending on the further analysis methods, the sections are mounted on special slides and dried for 10 min at room temperature.

### Cresyl Violet Staining

The entire staining procedure is carried out under RNase-free conditions. One day before staining, cresyl violet acetate (#7651.1, Carl Roth) is dissolved in 50% ethanol with stirring for several hours at room temperature, filtered through a sterile filter with 0.2 µm pore size (#83.1826.001, Sarstedt) and stored protected from light at room temperature. The further steps are performed in 50 ml Falcon tubes containing 30 ml of the required reagent. To optimize the RNA quality for each probe, the solutions have to be renewed every three slides (May-Zhang et al. 2018). Immediately before LMD, the thawed sections are fixed in 70% ethanol for 2 min. The sections are then coated with 0.5% cresyl violet solution for 20 s. Excess colour is briefly drained off on a Kimtech cloth. To differentiate the staining, the slides are repeatedly immersed in 50% and 70% ethanol for a few seconds until an optimal signal-to-background ratio of the staining is achieved. Subsequently, the samples are dehydrated in 100% ethanol for 30 s before drying the samples on the slide at room temperature.

### Laser Microdissection (LMD)

**Fig. 3:**
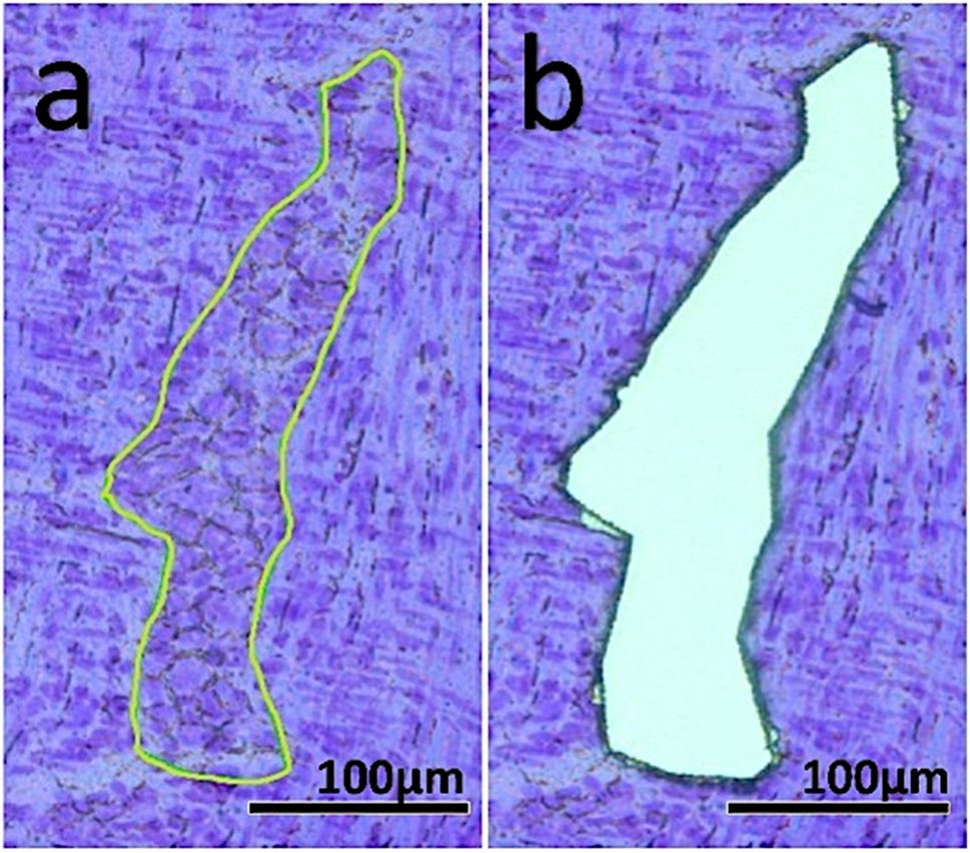
**a** 10 µm thick cryosection of rat jejunum (p30) stained with 0.5% cresyl violet solution. The precisely marked area (green line) contains 18,718 µm^2^ myenteric ganglia surrounded by the longitudinal and annular muscle layers. **b** High-precision section of the area without contamination by muscle cells using the Leica LMD6500. The lasered pieces are automatically collected in the lid of a 0.2 ml microtube

Laser microdissection can be used as a simple and fast method to isolate myenteric ganglia for RT-qPCR. For this method, up to ten 10 µm cryosections need to be mounted on RNase-free polyethylene naphthalate (PEN) membrane slides (1.4 mm) made for LMD (#11505151, Leica Microsystems) and lasered within one day or alternatively stored at − 80 °C. Following Leica LMD6500 system settings were applied: power 34; aperture 17; speed 33; specimen balance 18; × 20 magnification. In agreement with May-Zhang (2018), who describe 8 µm as the most suitable section thickness, we do not recommend cryosections thicker than 10 µm for optimal microdissection of the myenteric ganglia, as the process of cutting and mounting becomes significantly more difficult. The Leica LMD software is used to mark the myenteric ganglia to isolate areas between 1000 and 20,000 µm^2^ as seen in the p30 tissue example in Fig. 3a. Microdissecting larger areas is difficult as they often stick to the bottom of the slide and cannot be removed without causing artefacts. After cutting (Fig. 3b), the pieces fall directly into the lid of a 0.2 ml microtube. This method collects approximately 1 mm^2^ of sample to which 20 µl of RNA lysis buffer (Monarch® Total RNA Miniprep Kit (#T2010S, New England BioLabs)) is added. Before freezing at − 80 °C, the samples are centrifuged at 10,000 rpm for 5 min.

### RNA-Isolation

Total RNA from collected plexus myentericus samples is extracted using the Monarch® Total RNA Miniprep Kit (#T2010S, New England BioLabs). First, the LMD samples collected in 20 µl RNA system buffer are thawed on ice. Then 7 mm^2^ are pooled in a 1.5 ml microtube and up to 300 µl lysis buffer is added. According to the manufacturer's protocol for cells, samples are loaded onto the gDNA collection column and centrifuged at 16,000×*g* for 30 s. To optimize binding conditions for RNA, 300 µl ethanol (≥ 95%) is added to the flow. The mixture is then transferred to an RNA purification column and centrifuged; the flow-through is discarded. A DNase I treatment is performed on the column to enzymatically remove any remaining genomic DNA. The column is then washed three times with 500 µl RNA priming buffer and centrifuged according to the manufacturer's instructions. The column is then transferred to an RNase-free microfuge tube, 50 µl nuclease-free water is added directly to the centre of the column matrix and centrifuged for 30 s. The isolated RNA (~ 900 ng/µl) can be used directly for further processing or stored at − 80 °C. For purity check via RIN values, see Online Resource 1.

### cDNA Synthesis

For cDNA synthesis GoScript™ Reverse Transcription Mix, Oligo(dT) (#A2790, Promega) is used according to the manufacturer's protocol. 20 µl samples consisting of 4 µl nuclease-free water, 4 µl GoScript Reaction Buffer, Oligo(dT), 2 µl GoScript Enzyme Mix and 10 µl undiluted total RNA are incubated in 0.2 ml microvials for 5 min at 25 °C, 60 min at 42 °C and 15 min at 70 °C. Samples can then be stored at 4 °C for immediate RT-qPCR or alternatively stored at − 20 °C.

### RT-qPCR

The best results in RT-qPCR are obtained at cDNA concentrations of about 400 ng/µl. The expression levels for the housekeeping gene *GAPDH* (Primer sequence: 5′-ACT CCC ATT CTT CCA CCT TTG-3′, 3′-CCC TGT TGC TGT AGC CAT ATT-5′, Microsynth) are measured in triplicates and in at least three independent runs. On average, the following threshold cycle (C_t_) values p9 = 22.76 ± 0.98, p15 = 22.45 ± 0.76 and p30 = 22.71 ± 2.09, each with indicated standard deviation, were obtained. 10 µl GoTaq qPCR Master Mix (#A6001, Promega), 1.4 µl primer upstream, 1.4 µl primer downstream, diluted cDNA and ddH_2_O are used to a final volume of 20 µl. Using the CFX96 Real Time PCR Detection System (BioRad) samples are heated to 95 °C for 2 min, followed by 40 amplification cycles, 15 s at 95 °C and 60 s at 60 °C. Melting curves were recorded after each cycle and showed individual PCR products.

### Immunohistochemistry in the Tissue

Using this protocol, myenteric ganglia with neurons and ganglion cells can easily be visualized. The protocol works for different primary and secondary antibodies in cryosections of different ages, starting with p0 up to adults. For this purpose, tissue sections are fixed on Superfrost-Plus Adhesion Slides (#J1800AMNZ, Thermo Scientific) and stored at 4 °C. To limit the amount of antibodies required, the cryosections are first outlined with Pap Pen (#MKP-2, Kisker), fixed with 4% PFA in phosphate buffered saline (PBS) for 15 min and then rinsed with PBS for 3 × 5 min. For optimal antibody penetration, permeabilization of the cell membranes with 1% Triton-X-100 (#T8532; Sigma-Aldrich) in PBS for 15 min is performed. Then a thorough wash (3 × 5 min with PBS) is performed, before blocking non-specific binding sites by incubation with goat serum (#G9023, Sigma-Aldrich; 1:50 in PBS) for further 30 min. A short washing step with PBS for 2 min is followed by overnight incubation with the primary antibody at 4 °C. For visualization of myenteric neurons beta-III-tubulin (neuronal marker TUJ-1, #MAB1195, RD-Systems; 1:500 in PBS) or PGP 9.5 (#PA1- 10011, Thermo Fisher, 1:200 in goat serum) are used as specific antibodies. After two further washing steps with PBS (2 × 10 min), secondary antibodies, here e.g. goat anti-mouse (#T5393, Sigma-Aldrich; 1:750 in PBS) respectively goat anti-chicken IgY (#A11041, Invitrogen; 1:400 in PBS) are applied for 2 h.

This procedure can be repeated overnight at 4 °C for additional primary and secondary antibodies. Finally, a nuclear counterstain is performed with DAPI (#D9542. Sigma-Aldrich; 1: 5000 in PBS). Before the samples are finally embedded with fluorescence mounting medium (#S3023, Dako), intensive washing steps with PBS (3 × 5 min, 2 × 10 min) are performed.

**Fig. 4:**
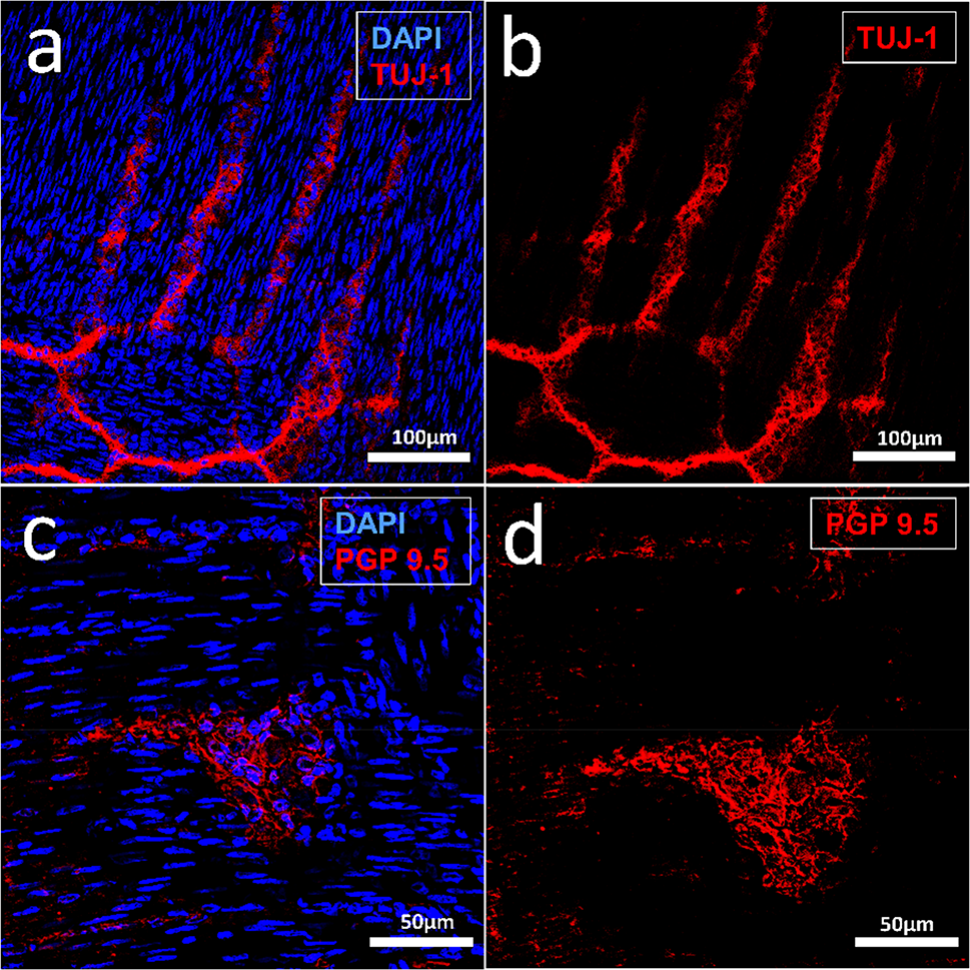
Confocal laser scanning microscopy of rat small intestine p15 (**a**, **b**) and p30 (**c**, **d**) cryosections. Between the longitudinal and circular muscle layers with horizontal and vertical elongated narrow cell nuclei (DAPI-positive, blue), the TUJ-1-positive neurons of the myenteric plexus (red) are visible. **c, d** PGP 9.5 labelled myenteric neurons (in c with nuclear counterstain, DAPI, blue)

Figure 4 shows an example of such staining for TUJ-1-positive neurons (red, a and b) and PGP 9.5 positive myenteric neurons (red; c and d) in cryosections of rat small intestine of p15 and p30.

## Cell Culture

### Reagents and Antibodies

Important note: all solutions must be prepared under sterile conditions at an appropriate workbench to avoid contamination with foreign substances or bacteria, fungi, etc. All solutions must be sterilized by membrane filtration (0.2 µm) or autoclaving.

- Dilution mix: 100 ml MEM-P/S: 99 ml MEM-Hepes (#M7278, Sigma-Aldrich); 1 ml PenStrep (PS) (#p4333, Sigma-Aldrich); store at 4 °C
- Enzymatic digestion mix: 1000 ml: 800 ml HBSS 1%PS (#HS264-500ML, Sigma-Aldrich); 150 ml Liberase (0.75 mg/ml); (#5401135001, Sigma-Aldrich); 50 ml DNAse (20 mg/ml = 2%); (#002139, CellSystems); store at − 20 °C
- Proliferation medium: For 50 ml: 47.875 ml Neurobasal medium (#10888022, Thermo Fisher); 0.125 ml Glutamine (#G7513, Sigma-Aldrich); 0.5 ml FBS (#F7524, Sigma-Aldrich); 0.5 ml PS (#P4333, Sigma-Aldrich); 1 µl bFGF (#SRP4039-50UG, Sigma-Aldrich); 20 ng/ml; 0.5 µl EGF (#SRP3238-100UG, Sigma-Aldrich); 10 ng/ml; 1 ml Neuromix 2a without retinoic acid (#12587010, Thermo Fisher); store at 4 °C
- Differentiation medium: For 50 ml: 47.375 ml Neurobasal medium (#10888022, Thermo Fisher); 0.125 ml Glutamine (G#7513, Sigma-Aldrich); 0.5 ml FBS (#F7524, Sigma-Aldrich); 0.5 ml PS (#P4333, Sigma-Aldrich); 1 ml Neuromix 2a plus retinoic acid (#17504044, Thermo Fisher); 0.5 ml Mitosis-inhibitor; store at 4 °C
- Mitosis-inhibitor stock solution: into 10 ml aqua dest. (1 mM/1 mM/1 mM) 2.46 mg 5-Fluoro-2′-Deoxyuridine (#21555, Serva); 2.8 mg Cytosine-ßD-Arabinofuranoside (#C6645, Sigma-Aldrich); 2.4 mg Uridine (#u3003, Sigma-Aldrich); store at − 20 °C
- Poly-d-Lysin (500 µg/ml) (#P7280-5MG, Sigma-Aldrich); store at − 20 °C

**Fig. 5:**
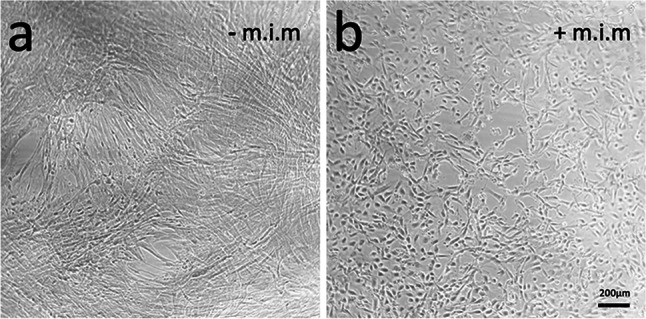
ENS neuron culture without (**a**) and with (**b**) mitotic inhibitor mix (m.i.m). Representative image on cells isolated from p15 tissue after 10 days in culture, with proliferation medium replaced by differentiation medium after 6 days. No passages were required

Figure 5 shows an example of an ENS neuron culture isolated from p15 tissue and cultivated for 10 days. On the left side, purified neurons were cultured without mitotic inhibitor, while the corresponding neurons on the right side were additionally treated with a mitotic inhibitor. While the neuron-typical branching is clearly visible in the image on the right, the cultures on the left are obviously overgrown by fibroblasts.

### Preparation

**Fig. 6:**
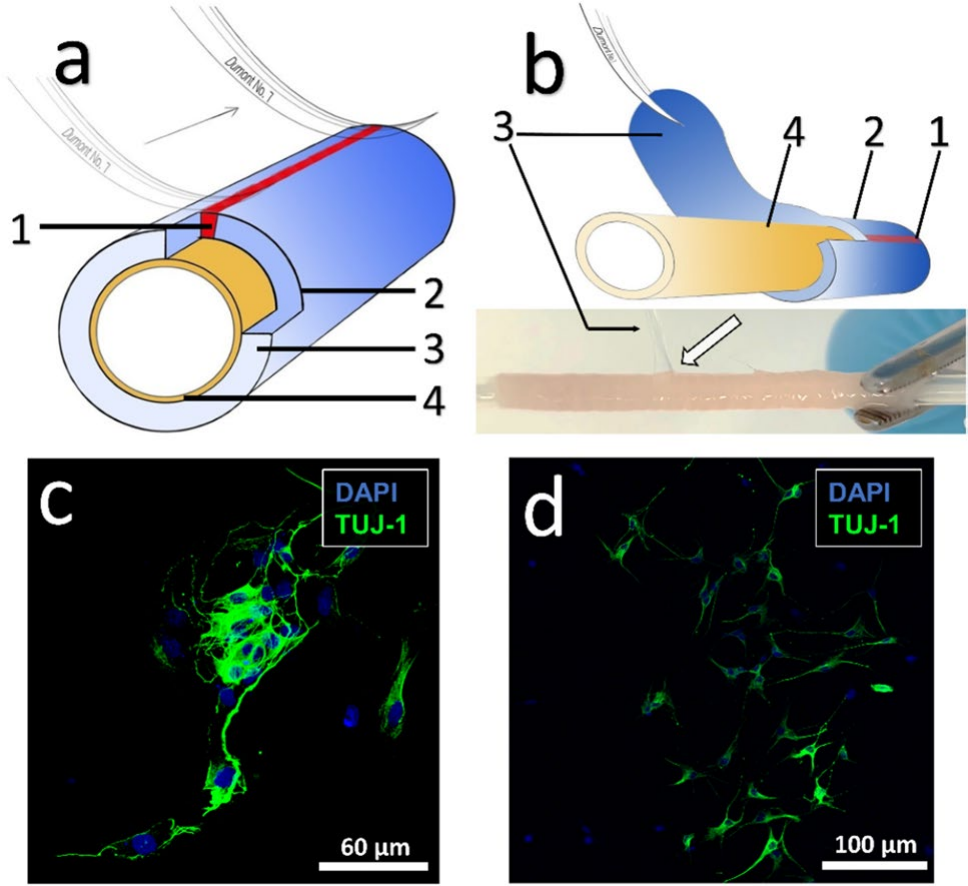
**a**, **b** Schematic representation of the intestinal tube with the removal of the muscularis for in vitro studies. The mesenterial line is marked in red (1) and embedded in the Tunica serosa (2) and Tunica muscularis drawn in blue (3). The inner tube in yellow represents the Tunica mucosa (4). **a** The muscularis is carefully separated along the mesenteric line with the forceps. **b** The separated Tunica muscularis (3) can now be gently removed from the underlying mucosa (4). Beneath is a picture of a stretched intestinal tube with the detached muscularis (and serosa), marked by the white arrow. Fluorescence microscopy of isolated myenteric plexus cells of p15 rat intestine after 9 (**c**) and 12 (**d**) days of cultivation. The difference in appearance is not due to the days of cultivation but shows the variations in which the cells grow. Neural marker beta-III-tubulin (TUJ-1)-positive neurons are shown in green

All work surfaces must be cleaned with NaOH-EDTA (0.1MNaOH, 1 mM EDTA) at the beginning. The preparation tools and Petri dishes are sterilized at 240 °C for 4 h. The intestines of Wistar rats (p15) are removed as previously described and stored in ice-cooled MEM during dissection. First, the intestines are cut into 3 cm long pieces, which are then pulled onto a Combitip Plus tip (#p1877, Eppendorf). The diameter of this tip is perfect for tissue preparation of p15 Wistar rats, as a slight pre-tensioning of the tissue is required for the removal of the tunica muscularis. Any parts of the mesentery that are still attached are now removed. The blunt edge of the forceps (#11271-30, Fine Science Tools) is then carefully moved across the mesenteric line to detach the muscularis muscles from the submucosal muscles without tearing the intestine apart (Fig. 6a). If this is done correctly, a second thin layer of tissue can be identified along the mesenteric line. This thin layer represents the muscularis, which is then grasped with another pair of sterile forceps and carefully detached (Fig. 6b). The proximal 5 mm around the stretched intestinal tube is released and the detached portion is then pulled to the distal end at the opposite point of the mesenteric line. It may be helpful to start the detachment process at one of the ends if the layers cannot be separated at the mesenteric line.

### Enzymatic Digestion

The isolated muscularis is cut into smaller pieces of 2 mm^2^ or less and transferred into a 1.5 ml tube (#72.690.001, Sarstedt) prefilled with 1.3 ml enzymatic digestion mixture. Depending on the age of the rat, it is incubated for 3.5–4.5 h at 37 °C in the Rollordrum (New Brunswick Scientific Co.). The best results were obtained with 3.5 h for p9, 4 h for p15, 4.5 h for p30. The tubes are shaken at least every hour to distribute the tissue evenly and thus providing more surface area for the digestion of smooth muscle cells. For our purpose, we used 15 ml Falcon tubes (#62.554.502, Sarstedt) to accommodate the 1.5 ml tubes and ensure smooth movement inside the Rollordrum. Following incubation, each tube is lightly shaken until the last remaining tissue dissolved. After centrifugation (Centrifuge 5430 R, Eppendorf) at 775 rpm and 37 °C for 5 min, the supernatant is discarded and the suspension containing the myenteric plexus cells from two tubes is transferred to a 35 mm Petri dish (#83.3900, Sarstedt). To further reduce the amount of enzyme mixture in the suspension, 2 ml of MEM-P/S is added. The dilution is allowed to stand for 1 min, then the supernatant is carefully removed. This procedure is repeated before 400 µl of proliferation medium is added for distribution into the 24-well plate (#83.3922, Sarstedt), combining up to 3 different Petri dishes.

### Cultivation

Before incubating the cell culture at 37 °C and 5% CO_2_, 500 µl of proliferation medium is added to each well; the medium is replaced after 3 days. After 6 days, the cells are transferred from the 24-well plate to a 12-well plate (#83.3921, Sarstedt) with a coverslip in each well. These coverslips were previously coated with Poly-d-Lysine (#P7280, Sigma-Aldrich) and incubated for 3 h at RT. To transfer the cells, the proliferation medium is discarded and replaced with 150 µl of differentiation medium. By scraping the bottom of each well with a pipette tip, the cells are detached and then transferred to the coverslips, combining two wells into one new well. In addition, 200 µl of differentiation medium is added to each well and completely replaced every 3 days.

Important note: 3 days after the start of differentiation in cell culture, experiments, e.g. the administration of factors, can be started.

### Immunohistochemistry in Cell Cultures

The coverslips are fixed with 4% PFA in PBS for 15 min and afterwards rinsed with PBS. To permeabilize the cell membranes, 0.3% Triton-X-100 (#T8532, Sigma-Aldrich) is applied in PBS for 15 min. To block non-specific binding sites, the coverslips are washed with PBS (3 × 5 min) and incubated with goat serum (#G9023, Sigma-Aldrich; 1:50 in PBS) for 30 min. After 2 min washing with PBS, primary antibody, here for example beta-III-tubulin (neuronal marker TUJ-1, #MAB1195, RD-Systems; 1:500 in PBS; Fig. 6c, d), is applied and incubated at 4 °C overnight. After washing with PBS (2 × 10 min), appropriate secondary antibodies (goat anti-mouse, #A11001, Molecular Probes; 1:1000 in PBS) are applied for 2 h, followed by further washing steps with PBS (3 × 2 min; 2 × 10 min). This is followed by a nuclear staining with DAPI (#D9542, Sigma-Aldrich; 1:5000 in PBS) for 30 min before intensive washing steps (3 × 2 min; 2 × 10 min with PBS). Finally, the coverslips are mounted in fluorescence embedding medium (#S3023, Dako).

## Discussion

There are many different techniques and protocols for studying the enteric nervous system. However, all of these have been performed independently in different species and at different ages, so that the instructions are not transferable to all applications without major adaptations. In order to perform a comprehensive study using neurons from the rat myenteric plexus, existing protocols had to be combined, refined and additional aspects and techniques had to be established. LMD is particularly suitable for analysis of myenteric neurons at the RNA level. It has already been shown several times that it can be used to isolate very precise high-quality RNA from the cells of interest (Funke 2011; Grover et al. 2012; Pieczora et al. 2017). However, to date, there is no detailed protocol for the preparation and LMD of myenteric cells from the rat small ileum. Existing protocols describe the LMD of intestinal cells from human intestine, which is easier to prepare due to its size (Böttner et al. 2010; Braun et al. 2018; May-Zhang et al. 2018). However, for functional studies, alternative protocols for model organisms such as the rat are essential.

Another important limitation of older protocols is the varying RNA quality of the samples, which can be partly explained by activated RNases as well as thermal effects during the process of lasering (Grover et al. 2012). To keep RNA quality as high as possible, an RNase-free, fast, clean and simple preparation technique is essential. In many gut preparation protocols, the mesentery is subsequently separated from the gut (May-Zhang et al. 2018). We were able to optimize the dissection by separating the mesentery directly from the bowel, leaving very little blood and fat on the bowel. This also saves considerable time and a labour-intensive dissection step. Due to the short dissection time, the tissue can be frozen within 15 min after decapitating the rat. Furthermore, this is done quickly and gently using the LIENS chamber, where several pieces are frozen at once. Flat frozen tissue is necessary to cut the cryosections with a high density of myenteric ganglia.

Following the protocol of Knott et al. (2003), the ileum of mice is embedded in freezing medium and frozen without a support, while May-Zhang et al. (2018) use dry ice and a flat support to freeze the human tissue. By alternatively using isopentane at exactly − 50 °C, we were able to further standardize this process. The reusable slides enclosed in aluminium foil as a base for the tissue are inexpensive and ensure safe and easy handling even after freezing. In addition, many devices in the laboratory are designed for slides, so standard cuvettes can be used, for example, to bake the slides in aluminium foil before preparation and allow the isopentane to evaporate in the freezer. The tissue can also be easily stored and transported in airtight and space-saving 50 ml tubes.

To ensure optimal RNA quality in the tissue, we recommend cresyl violet dissolved in ethanol (Herrfurth et al. 2017; May-Zhang et al. 2018). For staining cryosections, only a low concentration of 0.5% cresyl violet in distilled water is required. May-Zhang et al. (2018) recommend centrifugation of the cresyl violet solution before staining. The described method identifies and preserves myenteric ganglia in cryosections very well. The preparation techniques described are also suitable for methods of immunohistochemical labelling of proteins.

So far, however, most studies have used whole tissue preparations instead of cryosections from the relevant areas of the neural plexus (Pearson 1994; Tsai and Gariepy 2005). In these studies, the intestine was more elaborately prepared and subsequent freezing was not used. Since a large amount of tissue is stained, the amount of reagents, dye solutions or antibodies used is correspondingly larger.

Normally, the tissue to be sectioned on the cryostat is fixed with formalin and then infiltrated with 30% sucrose for several days to minimize freezing artefacts (Laranjeira et al. 2011; Pearson 1994). However, this time-consuming procedure is not suitable for LMD as it requires unfixed and freshly frozen tissue. In addition, storage in sucrose would be a constraint for our experiments as the tissue must be frozen flat and with the serosa facing downwards. Therefore, cryosections are fixed with 4% PFA immediately before immunohistochemical labelling.

The present protocol consisting of the detailed descriptions of small intestine preparation, cryosectioning, LMD and immunohistochemical labelling works very well in all age groups studied (p9, p15, p30) in the rat model system and is also transferable to the colon. There are just minor differences in the size and density of those plexus. However, it should be noted that especially for experiments with the small intestine of p0 rats, a correspondingly large number of animals is necessary to obtain enough ganglia for e.g. RT-qPCR, since fewer cryosections can be prepared from the intestine of younger rats. In addition, more smaller ganglia are found compared to older age. Otherwise, no differences in the appearance of the cryosections and cells can be seen. Immunohistochemical labelling of proteins in cryosections of the tunica muscularis, on the other hand, is easy to perform. Already cut samples can be stored unstained in the refrigerator at 4 °C for up to three months. Even then, the different cell types of the ENS and the surrounding muscularis can still be easily identified and assessed.

We have modified some major but also minor details compared to older protocols to improve the handling of myenteric cell cultures for older rats. Such protocols, involving new methods and optimizations, have so far focussed on mice or humans. To our knowledge, the highly specific isolation of the myenteric plexus ganglia in particular seems to be the essential step of a successful methodology. Here, the rapid and easy removal of the outer muscle layers and the precise enzymatic digestion of the dispensable cells is crucial. Although three different methods are currently used to obtain enteric neurons, the extraction of the intestine performed is very similar in different mammalian models. However, with the present protocol, we were able to reduce the time and difficulty of extracting the mesentery by cutting it just distal to the pyloric antrum and pulling the intestine through its own loops. This method results in a single fluid extraction process for the required length of bowel and self-extraction of the mesentery, which otherwise has to be performed in an additional, time-consuming step.

To culture enteric neurons, Lobo et al. showed that it is possible to extract just the desired portion of the intestine, followed by 30 min of trypsinization and incubation of all the detached cells (Lobo et al. 2007). Even though this procedure is relatively fast and easy, it results in a cell culture that depicts the composition of the entire intestine and not just enteric neurons. For studies that need a higher purity of enteric neurons other methods are more suitable. A more versatile method for isolating enteric neurons involves a longitudinal section along the mesenteric line in the desired intestinal segment, followed by manual separation of the outer muscle layer from the mucosa, as shown in adult mice (Grundmann et al. 2015). However, this method is time-consuming and requires some practice. To circumvent the drawbacks, our modified, simple method is, among other things, a modification of the protocol of Smith et al. (2013). By pulling the intestine onto a rod, the muscularis often detaches itself and is easy to remove, even for beginners. Using this method, up to six p15 and four p30 rats can be dissected per hour by two persons.

In the subsequent enzymatic digestion step, a liberase according to Grundmann et al. (2015) was used, which contains purified collagenase I and II and less aggressive proteases. Here, a prolonged digestion time while minimizing the amount of aggressive proteases such as caseinase leads to an increase in the amount of isolated myenteric ganglia. Compared to the protocol optimized for mouse intestines, for the digestion of rat intestines we increased the digestion time and used the Rollordrum to improve the digestion process through the continuous movement. Although this digestion method achieves a highly purified myenteric plexus culture, we still have to assume a low number of fibroblasts and smooth muscle cells to be in the cultures. Therefore, we supplemented the differentiation medium with a mitotic inhibitor mix, which is already established as a proliferation inhibitor for other rat neuronal cultures (Wessel et al. 2014). These modifications allowed us to keep the ENS neurons in culture for up to 12 days, which can be considered sufficient for many studies such as intoxication studies. However, in addition to the great advantages of the described protocol, the limitations must also be taken into account, particularly the fact that this protocol cannot be used to map the interaction of the cells in the tissue.

## Supplementary Information

Below is the link to the electronic supplementary material.

Supplementary file1 (MOV 91953 kb)

## Data Availability

All data generated or analysed during this study are included in this published article.
